# Lumefantrine and Desbutyl-Lumefantrine Population Pharmacokinetic-Pharmacodynamic Relationships in Pregnant Women with Uncomplicated Plasmodium falciparum Malaria on the Thailand-Myanmar Border

**DOI:** 10.1128/AAC.00267-15

**Published:** 2015-09-18

**Authors:** Frank Kloprogge, Rose McGready, Warunee Hanpithakpong, Daniel Blessborn, Nicholas P. J. Day, Nicholas J. White, François Nosten, Joel Tarning

**Affiliations:** a Centre for Tropical Medicine and Global Health, Nuffield Department of Clinical Medicine, University of Oxford, Oxford, United Kingdom; b Mahidol-Oxford Tropical Medicine Research Unit, Faculty of Tropical Medicine, Mahidol University, Bangkok, Thailand; c Shoklo Malaria Research Unit, Faculty of Tropical Medicine, Mahidol University, Mae Sot, Thailand; d WorldWide Antimalarial Resistance Network (WWARN), Oxford, United Kingdom

## Abstract

Artemether-lumefantrine is the most widely used antimalarial artemisinin-based combination treatment. Recent studies have suggested that day 7 plasma concentrations of the potent metabolite desbutyl-lumefantrine correlate better with treatment outcomes than those of lumefantrine. Low cure rates have been reported in pregnant women with uncomplicated falciparum malaria treated with artemether-lumefantrine in northwest Thailand. A simultaneous pharmacokinetic drug-metabolite model was developed based on dense venous and sparse capillary lumefantrine and desbutyl-lumefantrine plasma samples from 116 pregnant patients on the Thailand-Myanmar border. The best model was used to evaluate therapeutic outcomes with a time-to-event approach. Lumefantrine and desbutyl-lumefantrine concentrations, implemented in an *E*_max_ model, both predicted treatment outcomes, but lumefantrine provided better predictive power. A combined model including both lumefantrine and desbutyl-lumefantrine did not improve the model further. Simulations suggested that cure rates in pregnant women with falciparum malaria could be increased by prolonging the treatment course. (These trials were registered at controlled-trials.com [ISRCTN 86353884].)

## INTRODUCTION

Approximately half of the worlds' population is at risk for malaria. There were an estimated 584,000 deaths worldwide in 2013 (1). Children below 5 years of age and pregnant women have the highest risk of malaria infections and mortality (1). On the Thailand-Myanmar border, there are low seasonal transmission of Plasmodium falciparum malaria and high levels of antimalarial drug resistance. Malaria has been an important cause of maternal mortality and a major factor contributing to low birth weight. As a result of providing ready access to effective treatment and weekly antenatal visits to clinics, malaria-related maternal mortality rates in a stable population of displaced persons have fallen from an estimated 1,000 per 100,000 (430 to 2,320) in 1985 to zero from 2005 onwards (2). However, in migrants who have less access to health care, malaria-related maternal mortality has fallen less (2).

Artemisinin-based combination therapy (ACT) is the treatment of choice for uncomplicated Plasmodium falciparum malaria in adults and children and also during the second and third trimesters of pregnancy. The oral fixed-dose combination of artemether and lumefantrine (80 mg/480 mg given twice daily for 3 days) is the most commonly used ACT, providing excellent cure rates in nonpregnant patients (3–8). Artemether-lumefantrine is considered safe during the second and third trimesters of pregnancy, although variable cure rates in this population have been reported (9–11). High cure rates by day 42 were seen in pregnant women in Uganda (98.2%; range, 93.5 to 99.7%), but considerably lower cure rates were reported in pregnant women in Thailand, where transmission and thus host immunity is substantially lower (82.0%; range, 74.8 to 89.3%) (9, 10). A substantially smaller study in Tanzania reported lower cure rates in pregnant women (18% failures; 6/33 patients) than in nonpregnant women (5% failures; 1/22 patients) (11). More than 30% of the pregnant patients in both Thailand and Uganda had day 7 plasma lumefantrine concentrations (9, 10) below the previously defined target concentration of 280 ng/ml (12). Thus, the day 7 concentration of lumefantrine may not always correlate fully with cure rate, and variation might be due to ethnicity and/or regional differences as a consequence of lower background immunity and/or reduced susceptibility (13).

Lumefantrine's main metabolite, desbutyl-lumefantrine, has substantial antimalarial activity (14, 15). Mean half-maximal inhibitory concentrations (IC_50_s) of desbutyl-lumefantrine against clinical isolates of Plasmodium falciparum in three studies from northwest Thailand in 1997 and 1998 were 4.38, 6.54, and 8.94 nM, respectively (15). Geometric mean *in vitro* IC_50_s of desbutyl-lumefantrine for laboratory-adapted Plasmodium falciparum parasites were 9.0 nM for chloroquine-sensitive (3D7) and 9.5 nM for chloroquine-resistant (W2mef) cultures (14). Corresponding geometric mean IC_50_s for lumefantrine in the same *in vitro* experiments were more than five times higher: 65.2 nM and 55.5 nM for 3D7 and W2mef cultures, respectively (14). The mean *in vivo* ratio of lumefantrine to desbutyl-lumefantrine exposure was 27.4 [7.0 to 123] in Papua New Guinean children (14). Similar findings were reported for Colombian patients and pregnant patients in Thailand (8, 16). Thus, although the metabolite is intrinsically more potent, the predominant antimalarial effect is provided by the parent compound. Nevertheless, it has been suggested that efficacy and pharmacokinetic studies should include measurements of both lumefantrine and desbutyl-lumefantrine to enable the assessment of a combined parent-metabolite (lumefantrine/desbutyl-lumefantrine) drug effect (17).

The aim of this study was to assess the population pharmacokinetics and pharmacodynamics of lumefantrine and desbutyl-lumefantrine using a simultaneous drug-metabolite model in pregnant women in the second and third trimesters presenting with uncomplicated Plasmodium falciparum malaria in Thailand.

## MATERIALS AND METHODS

Pharmacokinetic data from pregnant patients who had undergone dense venous sampling (16) and those who had undergone sparse capillary sampling (18), as part of a larger efficacy trial (9) on the northwestern border of Thailand and Myanmar, were used for the current analysis. Pregnant patients were enrolled through weekly screening visits at the antenatal clinic (Shoklo Malaria Research Unit) (19). Initially, inclusion in the trial (9) was limited to women who had reappearance of Plasmodium falciparum after quinine monotherapy, the first-line treatment at the time, as artemether-lumefantrine had never been used to treat uncomplicated malaria in women during pregnancy. The pharmacokinetic and efficacy studies have been reported previously (9, 16, 18). After review of the first 20 delivered women in the study by the data safety monitoring committee, the study was then expanded to all patients with Plasmodium falciparum malaria in the second or third trimester. The study was approved by the Oxford Tropical Research Ethic Committee, the Ethics Committee of the Faculty of Tropical Medicine, Mahidol University, Bangkok, Thailand, and the Secretariat Committee on Research Involving Human Subjects of the World Health Organization.

Patients received standard treatment consisting of 80 mg artemether and 480 mg lumefantrine (Coartem; Novartis, Basel, Switzerland) twice daily for 3 days with 200 ml to 250 ml of chocolate milk (i.e., 6 g to 7 g fat). Frequent venous blood samples (2 ml) were drawn from 13 pregnant women before the last dose and at 0.5, 1, 2, 4, 6, 8, 12, 24, 48, 72, 96, 120, 144, and 168 h after the last dose (16). Sparse capillary blood from a finger prick was collected randomly from 103 pregnant women at 0 to 72 h, 72 to 96 h, 96 to 144 h, and 144 to 336 h after the first dose and on day 7 (18). Blood samples were centrifuged at 2,000 × g for 10 min, and the plasma was stored in cryotubes at −30°C or liquid nitrogen before transfer to −80°C.

Venous plasma concentrations of both lumefantrine and desbutyl-lumefantrine were quantified by using liquid chromatography with UV detection (HPLC-UV) (20). Capillary lumefantrine plasma concentrations were initially quantified by using HPLC-UV (20). All capillary samples were reanalyzed 5 years later for desbutyl-lumefantrine quantification using liquid chromatography-tandem mass spectrometry (LC-MS/MS). The lower limits of quantification for lumefantrine and desbutyl-lumefantrine were 24 ng/ml and 21 ng/ml, respectively, using HPLC-UV. For quantification of desbutyl-lumefantrine in capillary samples, a sensitive, specific, and rapid LC-MS/MS method for the determination of lumefantrine and its metabolite desbutyl-lumefantrine was developed and validated over concentration ranges of 9.7 to 20,000 ng/ml and 1 to 769 ng/ml, respectively, using 100 μl of plasma. After a simple solvent precipitation procedure, separation was achieved using an amide column (2.1 mm by 50 mm, 2.7 μm; Advanced Materials Technology, Wilmington, DE, USA) with a binary gradient solvent system consisting of 2.5 to 10 mM acetonitrile-ammonium acetate at pH 3.5. Mass detection was performed using a triple-quadrupole mass spectrometer (API5000; AB Sciex, Foster City, CA, USA) operating in positive electrospray ionization mode. The transition *m/z* of lumefantrine (528.2 to 276.1) and desbutyl-lumefantrine (472.1 to 346.1) was monitored using multiple-reaction monitoring. The method did not show any signs of severe ion suppression/enhancement for lumefantrine or desbutyl-lumefantrine. The within-day and between-day accuracy and precision at all quality control levels were below 7%. The coefficients of variation were lower than 8.2% and 7.9% for all quality control samples during clinical sample analysis using UV detection and mass spectrometric detection, respectively.

Molar units of lumefantrine and desbutyl-lumefantrine plasma concentrations were transformed into their natural logarithms and modeled simultaneously using a linear drug-metabolite model. Estimations and simulations were performed using NONMEM v.7.2 (ICON Development Solutions, Ellicott City, MD) with a G-Fortran compiler (Free Software Foundation, Boston, MA) on a Windows 7 operating system (Microsoft Corporation, Seattle, WA). Subroutine ADVAN6 was used for pharmacokinetic and pharmacodynamic model building, and the first-order conditional estimation method with interaction and the Laplacian estimation method with interaction were used to model the pharmacokinetic and pharmacodynamic data, respectively (21). Postprocessing and automation was done using Pearl-Speaks-NONMEM (PsN) v. 3.5.3 (22, 23), Xpose v. 4 (24), and R v. 2.15.1 (The R Foundation for Statistical Computing).

The objective function value (OFV; proportional to minus twice the log-likelihood of the data) was used to evaluate competing models during the model building process. A drop in OFV of 3.84 or more was considered a significant (*P* = 0.05) improvement between two hierarchical models after inclusion of one additional parameter (one degree of freedom). Goodness-of-fit diagnostics and physiological/pharmacological plausibility were also considered during the model building process.

A simultaneous population pharmacokinetic model for lumefantrine and desbutyl-lumefantrine was constructed. Different absorption (first-order and first-order with lag-time absorption), distribution (one-, two-, and three-compartment distribution), variability (between-subject variability) and residual error (additive, proportional, and combined additive and proportional errors on log-transformed data) models were considered. Model parameters were assumed to be log-normally distributed (equation 1), although a Box-Cox transformed distribution (equation 2) was also evaluated for relative bioavailability (25).

(1) $$P_{i}=P_{\text{TV}}\times e^{\eta }$$

(2) $$P_{i}=P_{\text{TV}}\times e^{\frac{e^{\eta^{\theta }-1}}{\theta }}$$

*P_i_* represents the individual parameter estimate, *P*_TV_ represents the typical parameter estimate for the population, η represents the between subject variability in the data, and θ represents the estimated Box-Cox transformation factor.

The differences in biological matrix (i.e., capillary versus venous data) might be explained partly by different quantification methods (i.e., HPLC-UV and LC-MS/MS). However, a correction factor embedded on a population level did not allow us to differentiate between differences resulting from the analytical quantification assay method and the biological matrix (for HPLC-UV, the lumefantrine venous and capillary matrix as well as the desbutyl-lumefantrine venous matrix; for LC-MS/MS, the desbutyl-lumefantrine capillary matrix) (13). For desbutyl-lumefantrine, it is even more complex to dissect the contribution due to potential stability issues over 5 years. The covariates body weight, estimated gestational age, and parasitemia were tried sequentially on all pharmacokinetic parameters using linear (equation 3), exponential (equation 4), and power (equation 5) relationships. Stepwise covariate modeling was applied using *P* values of 0.05 (ΔOFV = 3.84) and 0.001 (ΔOFV = 10.83) as cutoff criteria in the forward step and backward step, respectively.

(3) $$P_{i}=P_{\text{TV}}\times [1+\theta \times (\text{covariate}\;\text{value}-\text{median}\;\text{value})]\times {\text{e}}^{\eta }$$

(4) $$P_{i}=P_{\text{TV}}\times e^{\theta \times (\text{covariate value}-\text{median value)}}\times e^{\eta }$$

(5) $$P_{i}=P_{\text{TV}}\times {\left(\text{covariate}\;\text{value}/\text{median}\;\text{value}\right)}^{\theta }\times e^{\eta }$$

The best pharmacokinetic drug-metabolite model was fixed and used to evaluate the pharmacodynamic properties with a time-to-event approach. Constant (equation 6), Weibull (equation 7), and Gompertz (equation 8) hazard models were evaluated to describe recrudescent malaria.

(6) h(t)=λ

(7) $$h(t)=\alpha^{2}\times \lambda \times t^{\alpha -1}$$

(8) $$h(t)=\lambda \times e^{-t\;\times \;\frac{\ln(2)}{t_{1/2}}}$$

The hazard at a certain time point is described by *h*(*t*), λ represents the (baseline) hazard estimate, *t* represents the time, α represents the shape parameter in the Weibull distribution, and *t*_1/2_ represents the elimination half-life of the hazard in the Gompertz model.

Patients were screened weekly for malaria; and the start of the interval censoring time was therefore set to the time of the last parasite-negative visit, and the end of the interval was set to the time of the visit when recrudescent malaria was detected. This allows the event to take place at any time during the interval time and is not fixed to the time when the patients visited the clinic (equation 9).

(9) $$P(\text{event})=1-e^{-(\int h{\left(t\right)}_{\text{even}t^{-}}\int h{\left(t\right)}_{\text{start}\;\mathrm{int}erval})}$$

Lumefantrine and desbutyl-lumefantrine drug effects were evaluated using a sigmoidal *E*_max_ model (equation 10), where the slope factor (γ) was fixed to 1 and also estimated.

(10) $$E=E_{0}-\frac{C^{\gamma }\times E_{\max}}{{\text{EC}}_{50}^{\gamma }+C^{\gamma }}$$

Drug effects (*E*) were evaluated using individually predicted lumefantrine or desbutyl-lumefantrine plasma concentration (*C*), where *E*_0_ is the baseline effect (fixed to 1), *E*_max_ is the maximum effect (fixed to 1), and EC_50_ is the concentration realizing half of the maximum effect. Cumulative areas under the plasma concentration-time curves of lumefantrine or desbutyl-lumefantrine were also evaluated instead of predicted plasma concentrations. A stepwise covariate modeling procedure was performed on the pharmacodynamic parameters (i.e., baseline hazard and hazard half-life). Body weight, estimated gestational age, primary gravidity, age, parasitemia, and admission infection (i.e., malaria infection type at the start of the study; recrudescent or novel infection after previous treatment, or primary new infection) were tested as categorical covariates or as continuous covariates using an exponential relationship. *P* values of 0.05 and 0.001 were used as significance cutoff criteria during the forward and backward steps, respectively.

New malaria infections were modeled using a constant hazard model with interval censoring. The interval was based on observed parasitemia at detection and the back-calculated plausible interval of parasite release from the liver (hepatic schizogony) by using fixed high (10-fold increase in parasitemia every 48 h, with the assumption of 10^5^ parasites released from the liver) and low (5-fold increase in parasitemia every 48 h, 10^4^ parasites released from the liver) multiplication rates (26). Consequently, an event represents the appearance of asymptomatic malaria instead of the time of microscopy detection (symptomatic malaria), which could be potentially later due to travel time to the hospital and/or disregarding of the symptoms by the patient. Individually predicted lumefantrine and desbutyl-lumefantrine plasma concentrations were evaluated as modulators on the constant hazard function using a sigmoidal *E*_max_ model, with γ being estimated or fixed to 1.

The robustness of parameter estimates from the final model was assessed by calculation of relative standard errors and the 95% confidence intervals of parameter estimates from nonparametric bootstrap diagnostics (*n* = 1,000). The predictive power of the model was assessed by using visual predictive checks (27). For the pharmacokinetic data, simulations and observations were corrected for population predictions, and results were visualized by overlaying the 95% confidence intervals of the simulated (*n* = 2,000) 5th, 50th, and 95th percentiles with the 5th, 50th, and 95th percentiles of the observed data. The visual predictive check for the time-to-event analysis was visualized by overlaying the 95% confidence interval from the simulated (*n* = 2,000) events with the observed data. The visual predictive check for the relative hazard (compared to the baseline hazard) was visualized by overlaying the simulated (*n* = 2,000) 90% confidence interval with the estimated relative hazard and observed lumefantrine or desbutyl-lumefantrine plasma concentrations. The reliability of individual parameter estimates and goodness-of-fit plots were assessed by the calculation of eta and epsilon shrinkage (28).

Monte Carlo simulations (*n* = 2,000) were performed using the final population pharmacokinetic-pharmacodynamic model to assess the treatment outcome after a standard dose regimen (80 mg artemether/480 mg lumefantrine twice daily for 3 days), 80 mg artemether/480 mg lumefantrine twice daily for 5 days (18), and 80 mg artemether/480 mg lumefantrine twice daily for 10 days. The 10-day regimen was evaluated only in order to challenge the (full *E*_max_) model and would not be feasible in the clinic due to adherence issues. An increased and extended dose regimen (100 mg artemether/600 mg lumefantrine twice daily for 4 days; trial registry no. NCT01054248 [ClinicalTrials.gov]) was also evaluated, and for this simulation scenario, bioavailability was assumed to be reduced by 7.5% because of dose-limited absorption (5). Results were presented with box (25% to 75%) and whisker (2.5% to 97.5%) plots (GraphPad Software, Inc., CA, USA) for the simulated percentage of recrudescence-free (presumed cured) patients at day 42, day 7 lumefantrine plasma concentrations, and day 7 desbutyl-lumefantrine plasma concentrations.

## RESULTS

This pooled analysis was conducted using data from a previously reported study in pregnant women where dense venous (*n* = 13) and sparse capillary (*n* = 103) samplings were embedded in a large drug efficacy trial (Table 1) (16, 18). Seventeen of these 116 women had PCR confirmed recrudescent infections, 22 had novel infections during follow-up, and 77 had no parasite reappearance during the 42 days of follow-up or until delivery (Table 1). The median (range) time to recrudescent malaria was 23 (14 to 63) days. The median time to novel malaria infection was 35 (15 to 140) days (Table 1).

**TABLE 1:** Demographic summary of the study population^*a*^

Parameter	All data	Dense sampling (16)	Sparse sampling (18)
Study size	116	13	103
Number of PK samples (LF/DLF)	688/517	207/175	481/342
Number of PK samples per patient (LF/DLF)	5 (1–16)/4 (1–16)	16 (15–16)/16 (15–16)	5 (1–5)/4 (1–5)
Age (yr)	24 (14–42)	20 (14–42)	24 (15–42)
Body wt (kg)	49.0 (35.0–65.0)	47.0 (41.0–57.0)	49.0 (35.0–65.0)
Body temp (°C)	36.7 (35.0–39.3)	36.0 (35.0–38.5)	36.7 (35.0–39.3)
Estimated gestational age (weeks)	22.8 (13.1–39.0)	23.0 (13.1–38.0)	22.6 (13.1–39.0)
Primiparity (%)	23.4	31.0	22.3
Parasitemia (/μl)	3,260 (56.8–154,000)	837 (91–251,000)	4,400 (57.0–154,000)
No parasite reappearance (%)	66.4	92.3	63.1
New infections (%)	19.0	7.69	20.4
Time to new infections (days)	35 (15–140)	21	35 (15–140)
Recrudescent infections (%)	14.7	0	16.5
Time to recrudescent infections (days)	23 (14–63)		23 (14–63)

a Values are presented as medians (ranges) unless otherwise stated. PK, pharmacokinetics; LF, lumefantrine; DLF, desbutyl-lumefantrine.

**FIG 1:**
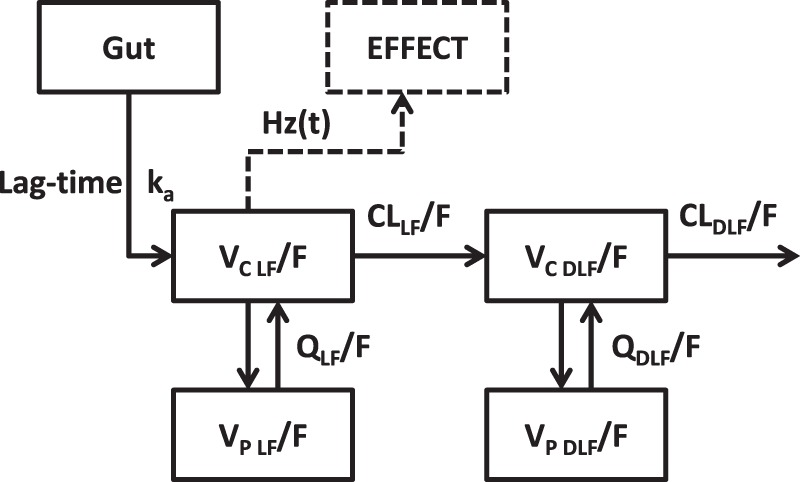
Visual representation of the structural population pharmacokinetic-pharmacodynamic model for lumefantrine/desbutyl-lumefantrine. LF, lumefantrine; DLF, desbutyl-lumefantrine; *k_a_*, absorption rate constant; *V_C_*/*F*, apparent central volume of distribution; *V_P_*/*F*, apparent peripheral volume of distribution; *Q*/*F*, intercompartmental clearance; CL/*F*, elimination clearance; and Hz(*t*), hazard function.

**FIG 2:**
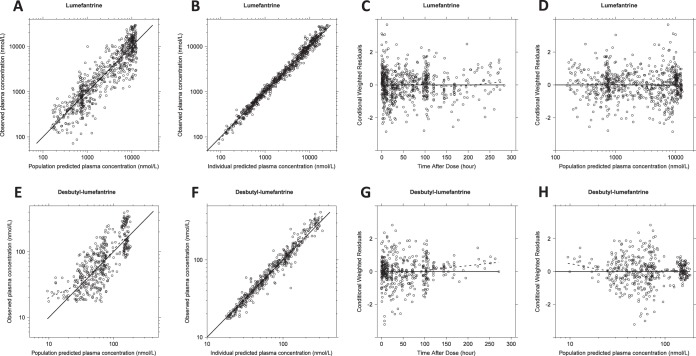
Goodness-of-fit diagnostics from the final pharmacokinetic model for lumefantrine (A to D) and desbutyl-lumefantrine (E to H). (A and E) Observed plasma concentration versus population predicted plasma concentrations; (B and F) observed plasma concentrations versus individual predicted plasma concentrations; (C and G) conditional weighted residuals versus time after dose; (D and H) conditional weighted residuals versus population predicted plasma concentration. The black solid lines, black dashed lines, and open circles represent the line of identity, the trend line, and observations, respectively. Two lumefantrine and desbutyl-lumefantrine baseline values were excluded for the diagnostic plots, as they were predicted to be 0.

A two-compartment drug-metabolite model using first-order absorption with lag time was used to describe simultaneously the lumefantrine and desbutyl-lumefantrine pharmacokinetic data (Fig. 1). The addition of additional distribution compartments did not result in a significant improvement in the model fit. Implementation of relative bioavailability significantly improved the model fit (ΔOFV = −284) and a Box-Cox transformation on relative bioavailability (ΔOFV = −4.86) was needed to correct for a systemic under prediction in the diagnostic plots (Fig. 2). A correction factor was implemented at a population level to correct for concentration differences between measurements in venous and capillary plasma (Table 2). Different random additive residual variability components were implemented for venous and capillary samples for both lumefantrine and desbutyl-lumefantrine (Table 2).

**TABLE 2:** Primary population parameter estimates from the final pharmacokinetic-pharmacodynamic model

Primary parameter^*a*^	Population estimate^*b*^ (% RSE^*c*^)	95% CI^*d*^	% CV for IIV^*b*^ (% RSE^*c*^)	95% CI^*d*^
*k_a_* (h^−1^)	0.0577 (7.93)	0.0526–0.0655		
Lag time (h)	1.31 (43.6)	0.0131–2.05		
*F* (%)	100 (fixed)		51.2 (14.3)	42.7–58.3
Box-Cox shape parameter on *F*	−0.394 (48.3)	−0.765 to −0.0279		
CL_LF_/*F* (liters/h)	5.35 (12.9)	4.11–6.77	11.2 (49.5)	3.20–15.7
*V*_C LF_/*F* (liters)	28.4 (26.8)	17.3–46.8	119 (29.4)	78.1–178
*Q*_LF_/*F* (liters/h)	1.55 (13.9)	1.17–2.00	23.9 (45.2)	9.14–33.7
*V*_P LF_/*F* (liters)	147 (13.9)	110–187		
CL_DLF_/*F* (liters/h)	197 (11.5)	156–245	23.2 (59.1)	6.93–36.5
*V*_C DLF_/*F* (liters)	6,490 (21.1)	3,460–8,970	90.5 (93.9)	44.8–181
*Q*_DLF_/*F* (liters/h)	250 (19.1)	183–369		
*V_P_*_DLF_/*F* (liters)	13,200 (12.6)	10,500–16,900		
LF capillary conversion factor	0.878 (13.2)	0.667–1.12		
DLF capillary conversion factor	0.464 (11.5)	0.371–0.585		
Parasitemia exponentially^*e*^ on CL_DLF_	0.133 (31.8)	0.0455–0.210		
EGA power^*e*^ on *k_a_*	−0.715 (19.1)	−0.972 to −0.474		
EGA linear^*e*^ on *Q*_LF_ (%)	−2.71 (22.8)	−3.59 to −1.34		
σ venous LF	0.252 (54.0)	0.0208–0.550		
σ capillary LF	0.0464 (15.4)	0.0332–0.0598		
σ venous DLF	0.335 (52.7)	0.0178–0.639		
σ capillary DLF	0.0326 (18.9)	0.0213–0.0458		

a LF, lumefantrine; DLF, desbutyl-lumefantrine; Box-Cox shape parameter, shape parameter on Box-Cox transformation; *k_a_*, absorption rate constant; *V_C_*/*F*, apparent central volume of distribution; *V_P_*/*F*, apparent peripheral volume of distribution; *Q*/*F*, inter-compartmental clearance; CL/*F*, elimination clearance; σ, variance of the unexplained residual variability.

b Population mean values and inter-individual variability (IIV) estimated by NONMEM. Coefficient of variation (% CV) for IIV is calculated as $100\times \sqrt{e^{\text{estimate}}-1}$ .

c Relative standard error (RSE) is calculated as 100 × (standard deviation/mean parameter estimate) from 1,000 iterations of a nonparametric bootstrap diagnostics.

d The 95% confidence interval (95% CI) is displayed as the 2.5 to 97.5 percentiles of the bootstrap estimates.

e Exponential covariate relationship is determined as exp{θ× [covariate − median(covariate)]}; power covariate relationship is determined as [covariate/median(covariate)]^θ^; linear covariate relationship is determined as 1 + {θ× [covariate − median(covariate)]}.

Body weight in a power relation with all clearance (exponent fixed to 0.75) and distribution (exponent fixed to 1) parameters did not improve the model fit. The covariates parasitemia (linear on lumefantrine elimination clearance, exponential on desbutyl-lumefantrine elimination clearance, and exponential on desbutyl-lumefantrine central apparent volume of distribution) and estimated gestational age (power relationship on the absorption rate constant, linear on lumefantrine intercompartmental clearance, exponential on lumefantrine peripheral apparent volume of distribution, exponential on desbutyl-lumefantrine central apparent volume of distribution, exponential on lumefantrine elimination clearance, linear on desbutyl-lumefantrine elimination clearance, and exponential on desbutyl-lumefantrine intercompartmental clearance) were selected during the forward step of the stepwise covariate modeling (*P* ≤ 0.05). However, only parasitemia on desbutyl-lumefantrine elimination clearance and estimated gestational age on the absorption rate constant and lumefantrine intercompartmental clearance could be retained in the backward step of the stepwise covariate modeling (*P* ≤ 0.001).

**FIG 3:**
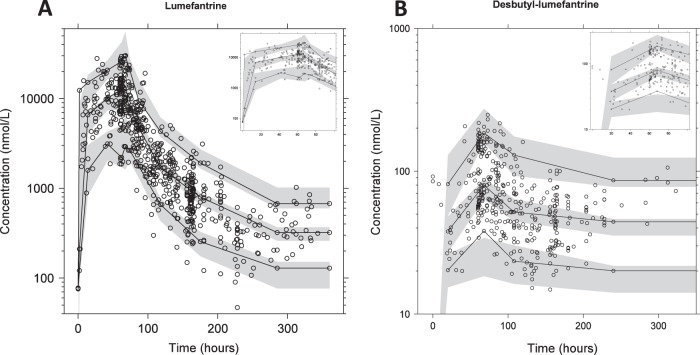
Prediction-corrected visual predictive checks (*n* = 2,000) for lumefantrine (A) and desbutyl-lumefantrine (B). The black circles represent the observations, the black solid lines represent the 5th, 50th, and 95th percentiles of the observed data, and the gray shaded areas represent the 90% confidence intervals of the simulated 5th, 50th, and 95th percentiles.

The basic goodness-of-fit plots showed accurate and precise predictions of the lumefantrine and desbutyl-lumefantrine concentrations (Fig. 2). Desbutyl-lumefantrine plasma concentration data below the limit of quantification in the terminal elimination phase did not result in any major model misspecification. The prediction-corrected visual predictive check (*n* = 2,000) indicated adequate predictive power for lumefantrine and desbutyl-lumefantrine plasma concentrations (Fig. 3). Eta shrinkage was high and ranged between 28.1% and 45.5%. Epsilon shrinkage was 4.76% for venous lumefantrine, 21.0% for capillary lumefantrine, 3.90% for venous desbutyl-lumefantrine, and 28.8% for capillary desbutyl-lumefantrine. Final model-derived pharmacokinetic parameter estimates and secondary estimates are presented in Tables 2 and 3, respectively.

**TABLE 3:** Secondary population parameter estimates

Secondary parameter	Median value (range)	
	Lumefantrine	Desbutyl-lumefantrine
Day 7 concn (ng/ml)	436 (81.2–1,650)	30.3 (7.55–91.8)
AUC_0-∞_ (h · μg/ml)	552 (114–1,340)	12.2 (3.18–37.3)
*C*_max_ (ng/ml)	6,660 (1,440–15,800)	73.2 (18.7–192)
*T*_max_ (h)	5.88 (2.50–13.1)	14.4 (9.07–42.4)
*t*_1/2_ (days)	3.65 (2.81–7.59)	4.08 (2.93–6.21)

Pharmacokinetic parameters were fixed to the final estimates, and pharmacokinetic-pharmacodynamic data were fitted simultaneously (Table 4). Interval censoring for the weekly screening was implemented for recrudescent malaria. A Gompertz hazard model performed better compared to a constant (ΔOFV = −8.00) or Weibull (ΔOFV = −10.0) hazard model. Both desbutyl-lumefantrine (ΔOFV = −5.19) and lumefantrine (ΔOFV = −7.17) plasma concentrations had a significant effect on the hazard of recrudescent malaria using an *E*_max_ model with the slope factor fixed to 1. However, lumefantrine contributed the larger effect, and an additive drug effect of desbutyl-lumefantrine did not further improve the model fit. Estimated gestational age (exponential on baseline hazard and hazard elimination half-life) and body weight (exponential on baseline hazard) were all selected as significant covariates during the forward addition step of the stepwise covariate modeling (*P* ≤ 0.05). However, none of these covariates could be retained in the backward elimination of selected covariates (*P* ≤ 0.001). Admission malaria type (i.e., recrudescent infection, novel infection, or primary new malaria) as a covariate on baseline hazard, hazard half-life, or lumefantrine IC_50_ did not significantly improve the model fit either. The median (range; quantile range) individually predicted lumefantrine concentrations at day 7 and area under the concentration-time curve from zero to infinity (AUC_0–∞_) in patients with recrudescent malaria on admission (431 [121 to 1,650; 303 to 545] ng/ml and 541 [167 to 1,330; 367 to 694] μg · h/ml) or novel malaria (439 [140 to 933; 307 to 528] ng/ml and 528 [244 to 892; 384 to 636] μg · h/ml) tended to be only slightly lower than in patients with admission primary new malaria infections (469 [158 to 1,210; 326 to 710] ng/ml and 595 [157 to 1,340; 474 to 804] μg · h/ml), but it did not reach statistical significance (*P* ≥ 0.05; analysis of variance [ANOVA] and regression analysis on logarithmically transformed day 7 and AUC).

**TABLE 4:** Time-to-event analysis

Parameter	Lumefantrine drug effect		Desbutyl-lumefantrine drug effect	
	Population estimate^*a*^ (% RSE^*b*^)	95% CI^*c*^	Population estimate^*a*^ (% RSE^*b*^)	95% CI^*c*^
Baseline hazard (recrudescent infections per week)	0.0845 (43.4)	0.0411–0.219	0.105 (36.7)	0.0496–0.223
Hazard half-life (h)	400 (23.9)	233–606	233–606	247–522
IC_50_ (ng/ml)	169 (44.0)	32.1–296	7.05 (43.9)	1.49–15.1

a Population mean values estimated by NONMEM.

b Relative standard error (RSE) is calculated as 100 × (standard deviation/mean parameter estimate) from 1,000 iterations of nonparametric bootstrap diagnostics.

c The 95% confidence interval (95% CI) is displayed as the 2.5 to 97.5 percentiles of the bootstrap estimates.

**FIG 4:**
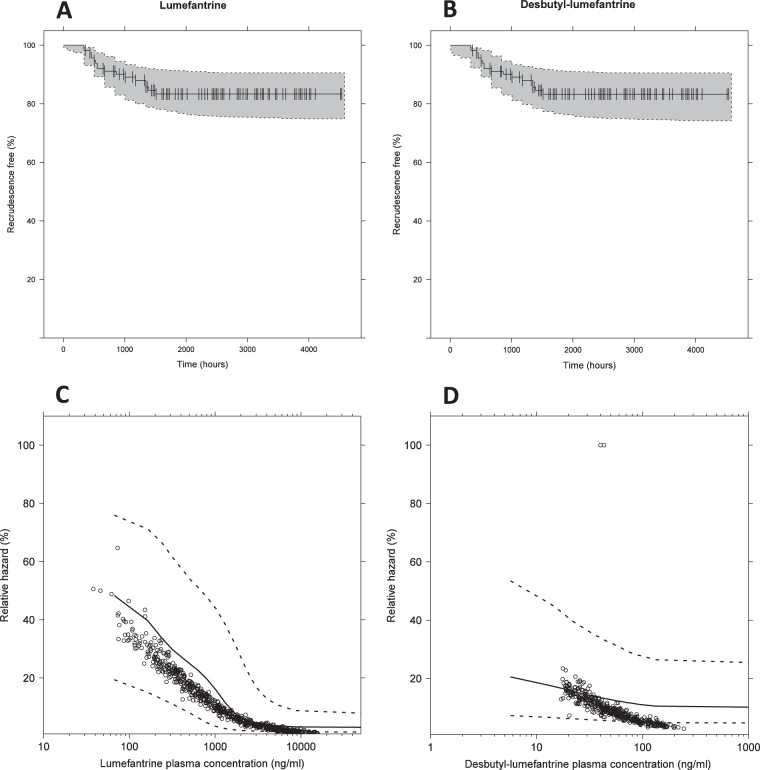
Visual predictive check (*n* = 2,000) for the time-to-event survival analysis with lumefantrine drug effect (A) and desbutyl-lumefantrine drug effect (B). Visual predictive check (*n* = 2,000) for relative hazard of recrudescent malaria compared to the baseline hazard versus lumefantrine plasma concentrations (C) and desbutyl-lumefantrine plasma concentrations (D). The open circles represent observed concentrations and corresponding estimated relative hazards, and the solid and dashed lines represent the median and the 5th and 95th percentiles of 2,000 simulations using the final pharmacokinetic-pharmacodynamic model.

The 90% confidence interval of 2,000 simulated Kaplan-Meier plots described the observed recrudescent malaria accurately using either lumefantrine or desbutyl-lumefantrine as drug effects (Fig. 4A and B). However, the 90% confidence interval of relative hazard versus plasma concentration indicated a better predictive power for the model using lumefantrine as a predictor of outcome than for that using desbutyl-lumefantrine (Fig. 4C and D).

Novel infections could be described using a constant hazard model with interval censoring, based on a back-calculated time interval representing the approximated start of the parasite blood stage. The implementation of a lumefantrine or desbutyl-lumefantrine drug effect (*E*_max_ and sigmoidal *E*_max_) on the constant hazard model did not improve the model fit significantly.

**FIG 5:**
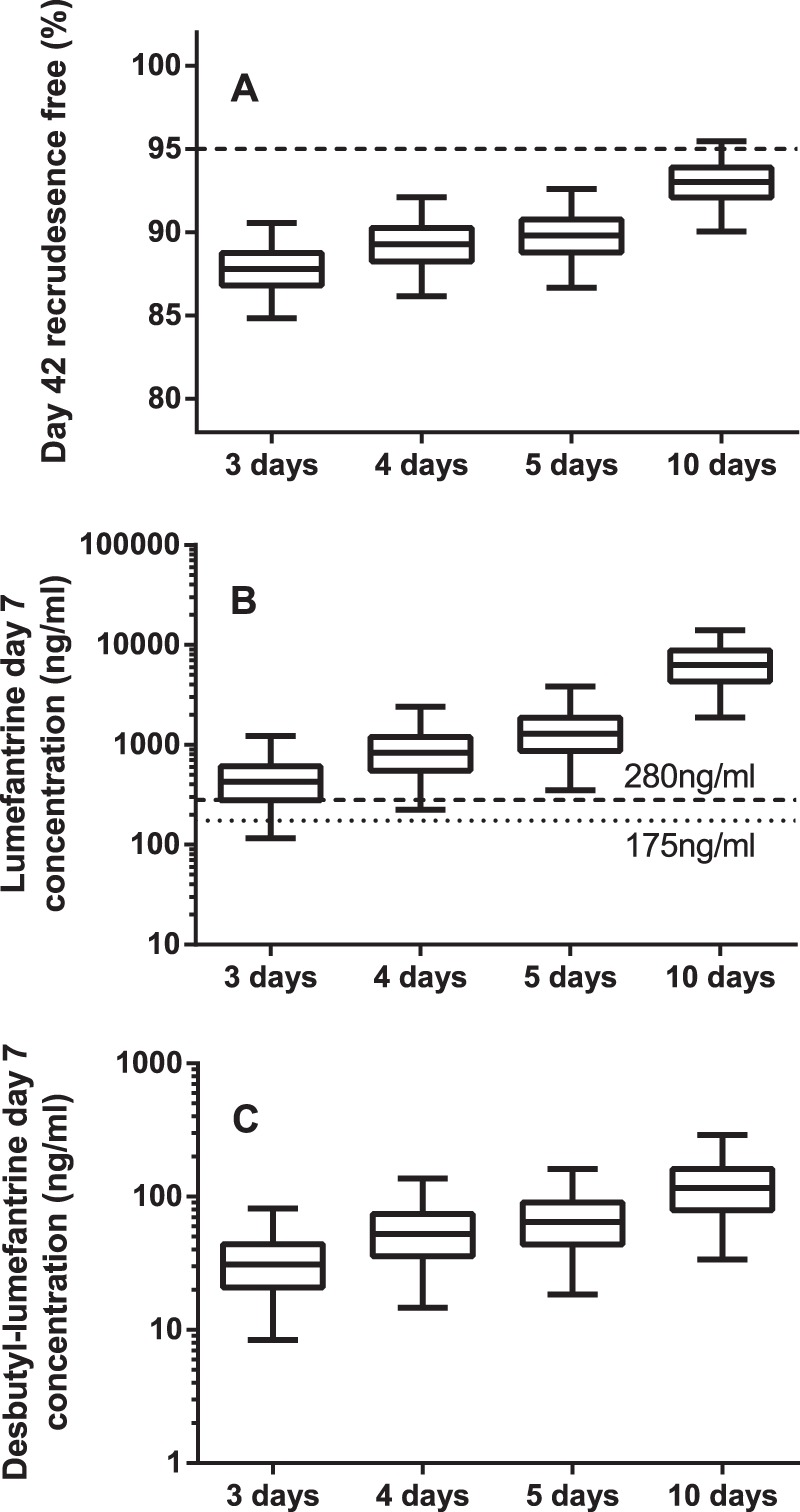
Dose optimizations with the population pharmacokinetic-pharmacodynamic model using a lumefantrine drug effect. Simulation scenarios were twice-daily dosing (4 tablets per occasion) for 3, 5, and 10 days and twice-daily dosing (5 tablets per occasion) for 4 days. (A) Percentage recrudescence-free patients at day 42; (B) lumefantrine plasma concentrations at 168 h; (C) desbutyl-lumefantrine plasma concentrations at 168 h. The boxes and whiskers in panel A to C represent the 25% to 75% and 2.5% to 97.5% percentiles of the simulated data. The horizontal dashed line in panel A represents the 95% recrudescent malaria-free patients. The two horizontal dashed lines in panel B represent the 280-ng/ml and 175-ng/ml target lumefantrine plasma concentrations (12, 35).

Monte-Carlo simulations (*n* = 2,000) were performed using the final pharmacokinetic-pharmacodynamic model for a twice-daily artemether-lumefantrine treatment (with four tablets at each occasion) for 3 days, 5 days, and 10 days and a twice-daily dosing regimen with 5 tablets per dosing occasion for 4 days. Not surprisingly, simulated lumefantrine and desbutyl-lumefantrine concentrations at days 7 and 14 and day 42 cure rates increased with extended treatment duration (Fig. 5).

## DISCUSSION

Artemether-lumefantrine is the most widely used artemisinin combination treatment and is also the most widely used in pregnancy. Artemether provides rapid parasite killing and therefore rapid recovery, whereas lumefantrine removes the parasites that remain after two parasite asexual cycles have been exposed to artemether. Lumefantrine is metabolized to an active metabolite, desbutyl-lumefantrine. In this large series of 116 pregnant patients studied on the Thailand-Myanmar border, lumefantrine and desbutyl-lumefantrine pharmacokinetic data were analyzed simultaneously. The developed model allowed characterization of the pharmacokinetic-pharmacodynamic relationship for both lumefantrine and desbutyl-lumefantrine.

### Pharmacokinetics.

Lumefantrine absorption and disposition pharmacokinetics were best described using first-order absorption with lag-time followed by a two-compartment distribution model (Fig. 1). This was identical to the previously performed pharmacokinetic analysis of the sparsely sampled capillary lumefantrine data (18). Desbutyl-lumefantrine was best described using two disposition compartments, as previously shown in Papua New Guinean children (17). Estimated gestational age as a covariate on intercompartmental clearance of lumefantrine corresponds with previously published findings in pregnant and nonpregnant women in Uganda, where pregnancy as a categorical covariate was a significant covariate in intercompartmental clearance (13). Estimated gestational age as a covariate on *k_a_*, resulting in a reduced *k_a_* with increasing gestational age, could be explained by decreased gut motility during pregnancy (29–31). Admission parasitemia correlated significantly with desbutyl-lumefantrine elimination clearance, resulting in a higher clearance for patients with higher parasitemia on admission. However, the mechanism underlying this covariate relationship is not known. The final population pharmacokinetic model showed a good predictive performance and goodness-of-fit diagnostics for lumefantrine and desbutyl-lumefantrine.

### Pharmacodynamics.

Pharmacokinetic parameters were fixed in the pharmacokinetic-pharmacodynamic model, and the chosen approach resulted in an adequate fit of observed data for recrudescent malaria (Fig. 4). Pharmacokinetic artemether and dihydroartemisinin measurements were available for only 13 patients in the dense-venous-sampling study arm and therefore not modeled separately and embedded in the overall pharmacokinetic-pharmacodynamic model. Furthermore, none of these 13 patients had recrudescent infections, although this could have been a result of lower parasitemia at admission, as these patients had recrudescent infections after quinine treatment. The pharmacokinetic-pharmacodynamic relationship between lumefantrine or desbutyl-lumefantrine and treatment outcome in the developed model therefore represents the sum of antimalarial activities of artemether, dihydroartemisinin, lumefantrine, and desbutyl-lumefantrine. However, recrudescent malaria is more correlated to partner drug exposure as previously described for dihydroartemisinin-piperaquine (32). An *E*_max_ model, with an estimated slope factor, for both lumefantrine and desbutyl-lumefantrine resulted in a significant improvement of the model fit but it was not considered superior to an *E*_max_ model, with the slope fixed to 1, due to the instability of the model (>100% different slope factors with different initial estimates). A possible explanation for the poor stability might be the small number of recrudescent malaria episodes (17 out of 116 observations) during the follow-up period. Lumefantrine and desbutyl-lumefantrine plasma concentrations could be used interchangeably as predictors for recrudescent malaria, which might be explained by the similarity in pharmacokinetic concentration-time profiles. However, desbutyl-lumefantrine concentrations were substantially lower, which explains the lower IC_50_ for desbutyl-lumefantrine than for lumefantrine. In this study, lumefantrine showed a better predictive power than desbutyl-lumefantrine, which might be a result of more accurate and precise predictions of lumefantrine than desbutyl-lumefantrine concentrations by the developed model (Fig. 4C and D). Therefore, lumefantrine concentrations were used in this model for *in silico* dose optimizations. Admission malaria type (i.e., recrudescent, novel, or primary new infection) did not improve the model fit significantly in this analysis. However, a previously published clinical analysis (9) showed a lower efficacy in women enrolled with recrudescent or novel infections compared to the women enrolled with primary new infections. Since the differences in lumefantrine concentrations between the groups were very small, other reasons, including different background immunity in these patients, cannot be excluded as possible explanations for this varying efficacy.

Although the study was not designed to evaluate the prophylactic effect of artemether-lumefantrine, neither lumefantrine nor desbutyl-lumefantrine plasma concentrations significantly suppressed novel infections in pregnant women on the Thailand-Myanmar border, an area of low and seasonal transmission. Lumefantrine has a relatively short half-life (3.3 days) and so exerts considerably less posttreatment prophylactic activity than mefloquine or piperaquine (33).

### Dose optimizations.

*In silico* dose optimizations displayed substantially increased lumefantrine exposures at day 7 and day 14 with the extended dose regimens. However, the relative improvement of the simulated cure rates at day 42 with extended treatments compared to the standard treatment was small. This could be explained by a failure to estimate a slope factor in the *E*_max_ model and/or the absence of the artemether/dihydroartemisinin drug effects in this model. Furthermore, relatively high eta shrinkage was observed, but it should not affect model mean predictions but only the simulated variability in the population. The model clearly underpredicted treatment success rates at day 42 after prolonged treatment, as the model does not adequately take into account the additional parasite killing provided by a longer course of artemether in this fixed combination. Prolonged artemether treatment will result in additional drug exposure in subsequent parasite life cycles, leaving a lower parasite density to be killed by lumefantrine. It is therefore important in future studies to dissect the artemether/dihydroartemisinin and lumefantrine/desbutyl-lumefantrine drug effects with respect to outcome to fully optimize the treatment. Indeed, a seven-day course of artemether alone provided 94% curative efficacy in earlier studies of recrudescent malaria in nonpregnant patients in this region, substantiating an increased killing of parasites when parasites are exposed to artemisinins for a sustained number of life cycles (34). A prolonged lumefantrine treatment will of course have similar effects, with a sustained killing of parasites in subsequent life cycles.

Simulations performed here of prolonged treatments using the developed model assumed a time-independent lumefantrine and desbutyl-lumefantrine pharmacokinetics. Previous studies have reported dose-limited lumefantrine absorption, where patients receiving a single daily lumefantrine dose for 3 days displayed 30% lower exposure than patients receiving the same total dosage split into two lumefantrine doses per day for 3 days (5). Therefore, a 7.5% decreased relative bioavailability was assumed for simulations of a 25% increased dosage (i.e., linearly extrapolated from 30% decreased bioavailability after a double dose). However, extending the dose regimen with additional days of dosing might result in additional alterations in the absorption of lumefantrine, and simulations should therefore be interpreted with caution.

Other factors rather than solely pharmacokinetic exposures might have an impact on the treatment outcome. For example, host immune responses are reduced during pregnancy, and this potentially means that a different lumefantrine day 7 concentration threshold is required in this special population. However, this is difficult to dissect and quantify, since ethnicity and geographical region might play a major role in the immune response. Furthermore, resistance to artemisinin derivatives in Southeast Asia increasingly compromises treatment efficacy, as this increases the number of residual parasites which have to be cleared by lumefantrine.

The most important issues at this point are to understand the underlying source of the reduced efficacy of artemether/lumefantrine during pregnancy on the Thailand-Myanmar border and to determine whether adequate dose optimization can decrease the risk of treatment failures and resistance development. This could be of great importance in anticipating a future scenario of reduced efficacy in Africa. Indeed, lower drug exposures and cure rates in pregnant women than in nonpregnant women have been shown in Tanzania, but cure rates remained high in Uganda even though drug exposures were also substantially lower during pregnancy (10, 11, 13). Overall, the lack of pharmacokinetic and efficacy studies in pregnant women in both Asia and Africa bring into question the dosing strategies in this group. Therefore, sufficiently powered studies with an equally sized nonpregnant control group and frequent pharmacokinetic (lumefantrine/desbutyl-lumefantrine and artemether/dihydroartemisinin plasma concentration) and pharmacodynamic samples (parasite clearance time and treatment outcome) are urgently needed. The continued use of the currently recommended doses of this drug combination in pregnant women might result in increased failure rates and the spread of drug-resistant parasites due to subtherapeutic lumefantrine levels.

In conclusion, a simultaneous drug-metabolite model was developed which adequately described the pharmacokinetic properties of lumefantrine and desbutyl-lumefantrine. The pharmacodynamic data were modeled using a time-to-event model linked to the pharmacokinetic model. Lumefantrine and desbutyl-lumefantrine could be used interchangeably as predictors of treatment failure, but lumefantrine realized a better predictive power in this model. An additive drug effect of desbutyl-lumefantrine did not further improve the model fit. The simulations based on the current lumefantrine/desbutyl-lumefantrine drug effect model, which contains substantial limitations, indicated that the drug combination of artemether and lumefantrine with the standard dosing regimen has suboptimal efficacy on the Thailand-Myanmar border in the treatment of uncomplicated Plasmodium falciparum malaria during the second and third trimesters of pregnancy and that increased treatment duration may facilitate improved cure rates.
